# Increased brain connectivity and activation after cognitive rehabilitation in Parkinson’s disease: a randomized controlled trial

**DOI:** 10.1007/s11682-016-9639-x

**Published:** 2016-10-18

**Authors:** María Díez-Cirarda, Natalia Ojeda, Javier Peña, Alberto Cabrera-Zubizarreta, Olaia Lucas-Jiménez, Juan Carlos Gómez-Esteban, Maria Ángeles Gómez-Beldarrain, Naroa Ibarretxe-Bilbao

**Affiliations:** 1 Department of Methods and Experimental Psychology, Faculty of Psychology and Education, University of Deusto, Bilbao, Biskay Spain; 2 OSATEK, MR Unit, Hospital of Galdakao, Galdakao, Basque Country Spain; 3 Neurodegenerative Unit, Biocruces Research Institute; Neurology Service, Cruces University Hospital, Bilbao, Biskay Spain; 4 Neurology Service, Hospital of Galdakao, Galdakao, Basque Country Spain

**Keywords:** Parkinson’s disease, Plasticity, Cerebral changes, Brain activation, Brain connectivity, Randomized controlled trial

## Abstract

Cognitive rehabilitation programs have demonstrated efficacy in improving cognitive functions in Parkinson’s disease (PD), but little is known about cerebral changes associated with an integrative cognitive rehabilitation in PD. To assess structural and functional cerebral changes in PD patients, after attending a three-month integrative cognitive rehabilitation program (REHACOP). Forty-four PD patients were randomly divided into REHACOP group (cognitive rehabilitation) and a control group (occupational therapy). T1-weighted, diffusion weighted and functional magnetic resonance images (fMRI) during resting-state and during a memory paradigm (with learning and recognition tasks) were acquired at pre-treatment and post-treatment. Cerebral changes were assessed with repeated measures ANOVA 2 × 2 for group x time interaction. During resting-state fMRI, the REHACOP group showed significantly increased brain connectivity between the left inferior temporal lobe and the bilateral dorsolateral prefrontal cortex compared to the control group. Moreover, during the recognition fMRI task, the REHACOP group showed significantly increased brain activation in the left middle temporal area compared to the control group. During the learning fMRI task, the REHACOP group showed increased brain activation in the left inferior frontal lobe at post-treatment compared to pre-treatment. No significant structural changes were found between pre- and post-treatment. Finally, the REHACOP group showed significant and positive correlations between the brain connectivity and activation and the cognitive performance at post-treatment. This randomized controlled trial suggests that an integrative cognitive rehabilitation program can produce significant functional cerebral changes in PD patients and adds evidence to the efficacy of cognitive rehabilitation programs in the therapeutic approach for PD.

## Background

Parkinson’s disease (PD) patients experience cognitive impairment in a wide range of cognitive domains (Goldman and Litvan 2011). Traditionally, PD has been related to deficits in executive functions, attention and visuospatial abilities, but also memory deficits are present in PD (Chiaravalloti et al. 2014; Whittington et al. 2006). Indeed, some studies found that memory was the most frequently affected cognitive domain in PD (Aarsland et al. 2010; Yarnall et al. 2014). This cognitive decline has been identified as a predictor of PD dementia and magnetic resonance imaging (MRI) studies have demonstrated a relationship between cognitive impairment and patterns of neurodegeneration in PD (Biundo et al. 2013; Christopher and Strafella 2013; Ibarretxe-Bilbao et al. 2011a).

Cognitive rehabilitation is a behavioral treatment for cognitive impairment based on the restoration, compensation and optimization of the cognitive functions that targets cognitive skills, but also improves daily functioning (Bahar-Fuchs et al. 2013; Wykes and Spaulding 2011). The efficacy of cognitive rehabilitation programs has been recently demonstrated in PD, showing improvements in cognitive functions (Hindle et al. 2013; Leung 2015; Pena et al. 2014) and functional disability (Pena et al. 2014).

Moreover, in the last few years, cognitive rehabilitation has been related to functional cerebral changes in other pathologies such as multiple sclerosis (Chiaravalloti et al. 2012; Filippi et al. 2012; Leavitt et al. 2014), mild cognitive impairment (Belleville et al. 2011), Alzheimer’s disease (van Paasschen et al. 2013) and schizophrenia (Penadés et al. 2013). Literature about structural cerebral changes associated to cognitive rehabilitation programs in neurodegenerative disorders is scarce. One study in multiple sclerosis found no significant white matter (WM) changes after cognitive rehabilitation (Filippi et al. 2012) but in patients with schizophrenia, increased WM was found after a 4 month-cognitive rehabilitation program (Penadés et al. 2013). Another study found grey matter (GM) preservation in schizophrenia patients after a 2-year intensive cognitive rehabilitation (Eack et al. 2010). However, to date, few studies have sought to elucidate cerebral changes associated with cognitive rehabilitation in PD. One study (Cerasa et al. 2014) found increased resting-state functional cerebral activation after attention rehabilitation in the left dorsolateral prefrontal cortex and the superior parietal cortex. In contrast, Nombela et al. (2011) found reduced brain activation during Stroop task after Sudoku training in PD. These two studies in PD patients included a specific treatment focused on the rehabilitation of one cognitive function and little is known about the neurobiological effects of an integrative cognitive rehabilitation program in PD, assessed with MRI combining both structural and functional MRI (fMRI) techniques.

In a previous study we demonstrated the efficacy of an integrative cognitive rehabilitation program, the REHACOP, on improving cognition and functional disability in PD patients (Pena et al. 2014). The objective of the present study was to assess the structural and functional cerebral changes associated to cognitive rehabilitation in the same cohort of PD patients. Due to the relevance of memory deficits in PD, a memory fMRI paradigm was included in this study to assess whether a cognitive rehabilitation program could produce changes in brain activation during learning and recognition memory tasks. Based on the findings of previous neuroimaging studies in neurodegenerative diseases (Belleville et al. 2011; Cerasa et al. 2014; Chiaravalloti et al. 2012; Filippi et al. 2012; Leavitt et al. 2014; Nombela et al. 2011; van Paasschen et al. 2013), we hypothesized that PD patients would show functional but not structural cerebral changes after attending REHACOP program compared with the control group (CG).

## Methods

### Subjects

The sample included 44 PD patients recruited from the Department of Neurology at the Hospital of Galdakao and from the PD Biscay Association (ASPARBI). PD patients were enrolled in the study if they fulfilled the UK PD Society Brain Bank diagnostic criteria. Other inclusion criteria were: i) age between 45 and 75; ii) Hoehn and Yahr disease stage ≤3 (Hoehn and Yahr 1998); iii) Unified PD Rating Scale (UPDRS) (Martinez-Martin et al. 1994) evaluated by the neurologist. Exclusion criteria were: i) the presence of dementia as defined by the DSM-IV-R (American Psychiatric Association 2003) and the Movement Disorders Society clinical criteria for PD-dementia; ii) scores on the Mini Mental State Examination <24; iii) the presence of other neurological illness/injury (traumatic brain injury); iv) unstable psychiatric disorders (e.g. schizophrenia); v) visual hallucinations as assessed by the Neuropsychiatric Inventory Questionnaire (Kaufer et al. 2000); vi) patients with depression evaluated with the Geriatric Depression Scale (score of >5) (Yesavage and Sheikh 1986). For the MRI part of the study, further exclusion criteria were: vii) other conditions incompatible with optimal pre-processing of MRI data and whole-group analysis such as cerebral haemorrhage, traumatic brain injury, dilated ventricles.

**Fig. 1:**
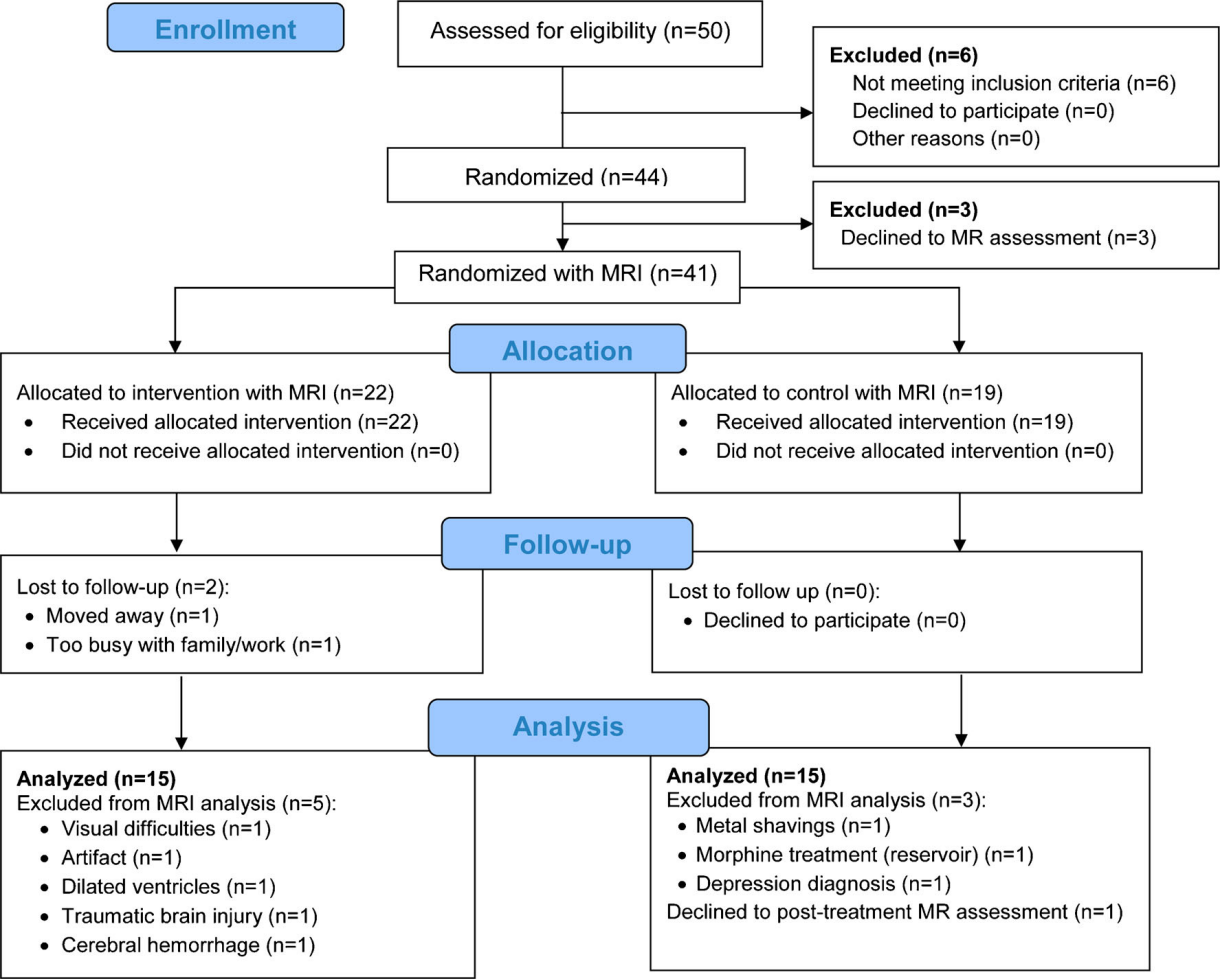
CONSORT Flow Diagram. CONSORT = Consolidated Standards of Reporting Trials; MRI = Magnetic Resonance Imaging

From the initial sample of 44 PD patients, three patients refused to attend MRI acquisition, two were lost to follow-up, eight patients were excluded from the MRI analysis and one refused to post-treatment MRI assessment (see Fig. 1 for the flow diagram). Hence, MRI analyses were carried out on 15 patients in the REHACOP group (patients receiving cognitive rehabilitation) and 15 patients in the CG, which received occupational therapy with the same duration and frequency.

Participants were symptomatically stable and evaluated during the “ON” period. Their Levodopa equivalent daily dose (LEDD) was registered (Tomlinson et al. 2010). The clinical and sociodemographic characteristics of the sample are shown in Table 1.

**Table 1:** Sociodemographic, clinical characteristics and behavioral data at baseline

	**REHACOP group (** ***n*** **= 15) Mean (SD)**	**CG (n = 15) Mean (SD)**	**U /** ***X*** ^**2**^	***p***
**Age**	66.20 (4.99)	67.60 (7.39)	98.00	.545
**Gender (Male)**	8 (53.3 %)	10 (66.7 %)	.13	.709
**Years of education**	11.40 (4.56)	10.13 (5.12)	97.50	.530
**Disease duration (years)**	6.13 (5.23)	8.41 (6.57)	84.00	.234
**Hoehn-Yahr stage**	1.90 (.28)	2.03 (.51)	4.06	.398
Stage 1	1	1		
Stage 1.5	1	2		
Stage 2	13	9		
Stage 2.5	0	1		
Stage 3	0	2		
**UPDRS Motor score**	19.27 (7.95)	25.93 (11.38)	75.00	.119
**LEDD**	631.32 (415.43)	988.15 (613.11)	73.00	.101
**NPI-Q**	4.47 (5.20)	3.13 (3.11)	106.00	.784
**MMSE**	27.93 (1.10)	26.56 (3.46)	102.50	.671
**Memory fMRI Paradigm: Behavioral data**				
Hits	9.73 (4.46)	9.71 (3.58)	94.50	.643
Correct Rejections	12.00 (2.87)	11.71 (3.12)	98.50	.772
False Negatives	5.13 (4.38)	5.21 (3.59)	94.00	.627
False Positives	2.87 (2.99)	3.21 (2.94)	95.00	.657

*REHACOP group* group receiving cognitive rehabilitation program, *CG* control group, *SD* Standard deviation, *UPDRS motor score* Unified Parkinson’s disease Rating Score, *LEDD* Levodopa Equivalent Daily Dose, *NPI-Q* Neuropsychiatric Inventory Questionnaire, *MMSE* Mini Mental State Examination

### Procedure

Participants underwent a neuropsychological assessment and MRI acquisitions at baseline and after treatment. After first evaluation, PD patients were randomly divided into REHACOP group and CG. Design details of this randomized controlled trial are as described in a previous report (Pena et al. 2014) which is registered in clinicaltrials.gov with number: NCT02118480.

### Intervention

The REHACOP is an integrative program which trains both basic and social cognition, in addition to psychoeducation, with mainly although not exclusively, bottom-up tasks. The REHACOP program was administered over three months, three times per week and one hour per day. Participants attending REHACOP group trained: attention (4 weeks; sustained, selective, alternant and divided attention), memory (3 weeks; verbal and visual learning, recall, and recognition), language (2 weeks; verbal fluency, synonyms, antonyms, definition of words and extract the main idea from text), executive functions (2 weeks; cognitive planning, verbal reasoning) and social cognition (1 week; moral dilemmas, empathy, theory of mind). Groups were made of 6–8 patients maximum and were conducted by two neuropsychologists. More information about the REHACOP program can be found in previous publication in PD (Pena et al. 2014). CG attended occupational therapy during the same period and frequency, and the activities included drawing, reading the daily news, and constructing with different materials (such as paper or wood).

### Neuroimage acquisition

Functional and structural imaging data were acquired on a 3 T MRI (Philips Achieva TX) at OSATEK, Hospital of Galdakao. All sequences were acquired during a single session.

T1-weighted images acquisition were obtained in a sagittal orientation (TR = 7.4 ms, TE = 3.4 ms, matrix size = 228 x218mm; flip angle = 9°, FOV = 250x250x180mm, slice thickness = 1.1 mm, 300 slices, voxel size = 0.98 × 0.98 × 0.60 mm, acquisition time = 4′55″).

Diffusion-weighted images were obtained, in an axial orientation in an anterior-posterior phase direction using a single-shot EPI sequence (TR = 7540 ms, TE = 76 ms, matrix size = 120x117mm; flip angle = 90°, FOV = 240x240x132mm, slice thickness = 2 mm, no gap, 66 slices, voxel size = 1.67 × 1.67 × 2.0 mm, acquisition time = 9′31″) with two identical repetitions (32 uniformly distributed directions b = 1000 s/mm^2^ and 1 b = 0 s/mm^2^).

The resting-state fMRI was obtained in an axial orientation in an anterior-posterior phase direction using sequence sensitive to blood oxygen level dependent (BOLD) contrast and multi-slice gradient echo EPI sequence (TR = 2100 ms, TE = 16 ms, matrix size = 80x78mm, flip angle = 80°, FOV = 240x240x130mm, slice thickness = 3 mm, 214 slices, voxel size = 3.00 × 3.00 × 3.00 mm, acquisition time = 7′40″).

Finally, patients also performed a memory fMRI paradigm inside the scanner. The fMRI images were acquired using a multi-slice gradient echo EPI sequence [TR = 2000 ms, TE = 29 ms, matrix size = 100x100mm, flip angle = 90°, FOV = 240x240x136mm, slice thickness = 3 mm; 280 slices (140 slices each learning and recognition task), voxel size = 1.67 × 1.67 × 3.00 mm, acquisition time = 9′36″ (4′48″ each learning and recognition task)].

The memory fMRI paradigm was presented with visual digital MRI-compatible high resolution stereo 3D glasses and Presentation® version 10.1 (Neurobehavioral Systems) running on Windows XP. The entire experiment consisted of a 10-block paradigm (learning and recognition tasks) that alternated activation and control conditions (5 blocks each). Each paradigm had a total duration of 280 s (28 s/block). Participants were also given a response box that recorded their behavioral responses. During the learning memory fMRI task, participants viewed 30 words (duration of 2 s per word and an inter-word interval of 1 s) and were asked to press the right button if they liked the word or the left button if they did not like the word. This task was used to ensure that the participants fixed their attention on reading the words as suggested by (Marsolek et al. 1992). During the recognition memory fMRI task, participants were asked to recognize words from a list of 30 words, of which 15 words had been presented during the learning memory fMRI task and 15 words were new. Participants were asked to press the button using their right hand to indicate if they remembered having read the word in the list during the learning fMRI task or the left button if they had not seen it before. In the control blocks, participants were presented with six combinations of letters (simulating the length of a word) of which three were the letters “AAAAAA” and the other three were random letters. Again, participants were asked to press the right button on the response box to indicate that the item was “AAAAAA” and press the left button when other combinations of letters appeared. This paradigm has previously been used and has demonstrated to show cerebral activation related to recognition memory in PD (Ibarretxe-Bilbao et al. 2011b; Lucas-Jiménez et al. 2015). Behavioral data were coded as “Hits” when participants answered yes and the answer was yes; “Correct rejections” when participants answered no and the answer was no; “False positives” when participants answered yes and the answer was no; and “False negatives” when participants answered no and the answer was yes. Two equivalent versions of this memory fMRI paradigm were used at both time points (pre- and post-treatment) in order to avoid learning effects. In the pre-treatment version, the words were four to six letters in length and of moderate frequency of use and were obtained from the Lexesp-Corco database. The post-treatment version was created including different words but with phonetic similarities and with the same number of syllables. Behavioral data from the recognition memory fMRI task were extracted and analyzed in SPSS.

### Neuroimage pre-processing

#### GM

Voxel-based morphometry (VBM) (Douaud et al. 2007) analysis were carried out using the FMRIB Software Library (FSL) tools (Smith et al. 2004). First, a study-specific template was created so that all of the images could be registered in the same stereotactic space (spatial normalization Then, the GM images were affine registered to the GM MNI-152 template and averaged to create an affine GM template. Next, the GM images were re-registered to this affine GM template using a non-linear registration and averaged to create a study-specific, non-linear GM template in standard space. Second, individual GM images were registered non-linearly to the study-specific template. After normalization, the resulting GM images were modulated by multiplying by Jacobian determinants to correct for volume change induced by the nonlinear spatial normalization. Then, the images were smoothed with a sigma of 3.5 mm (8 mm FWHM). Finally, cluster-based analyses were performed.

Cortical Thickness changes were analyzed with Freesurfer (Fischl 2012) (version 5.3; available at http://surfer.nmr.mgh.harvard.edu). The processing of T1 high-resolution images for the cortical surface reconstruction followed the freesurfer analysis pipeline (Dale et al. 1999; Fischl et al. 1999): Automated Talairach transformation, intensity normalization, skull stripping, WM segmentation, tessellation of the GM/WM boundary, automated topology correction, and surface deformation following intensity gradients to optimally place the fluid borders (GM/WM and GM/cerebrospinal fluid) at the location. All surface models were visually inspected for accuracy. No model was excluded due to misclassification of tissue types. Cortical thickness was calculated as the closest distance from the GM/WM boundary to the GM/cerebrospinal fluid boundary at each vertex on the tessellated surface. The bilateral mean cortical thickness values were extracted based on the parcellation of (Destrieux et al. 2010) and were introduced in SPSS for statistical analysis.

#### WM

Diffusion data were also preprocessed and analysed using FSL. First, each subject’s images were concatenated and radiologically oriented. Then, the data were corrected for motion and eddy currents, performed brain-extraction BET, and the diffusion gradients (bvecs) were rotated to be corrected accordingly, providing a more accurate estimate of tensor orientations (Jones and Cercignani 2010). Then, all fractional anisotropy (FA), mean diffusivity (MD), radial diffusivity (RD) and axial diffusivity (AD) images were obtained by fitting a tensor model to the raw diffusion data using FDT (DTIFIT). After, tract-based spatial statistic (TBSS) (Smith et al. 2006) was used for group comparisons. Using TBSS, the data were prepared to apply a nonlinear registration of all FA images into standard space, the mean FA image was created using a threshold of 0.2 and thinned to create a “mean FA skeleton” which represents the centres of all tracts common to the group. MD data were analysed using “tbss non FA” script from TBSS, which applies the original non lineal registration to the MD data, merges all subjects warped MD data into a 4D file, then project this onto the original mean FA skeleton, and creates the 4D projected data. The same process was repeated for RD and AD.

#### Resting-state fMRI

Resting-state fMRI data were acquired during a so-called resting-state block. Subjects were instructed to neither engage in any particular cognitive nor motor activity, to keep their eyes closed without thinking about anything in particular and they were told they could not fall asleep. Once the resting-state fMRI acquisition finished, the neuroradiologist talked with the patients and asked them whether they fell asleep or not. No patient reported to fall asleep. Foam padding and headphones were used to limit head movement and reduce scanner noise for the subject.

Functional connectivity analysis was performed using Conn Functional Connectivity Toolbox 14.p (Whitfield-Gabrieli and Nieto-Castanon 2012). First, each subject’ 214 functional images were realigned and unwraped, slice-timing corrected, coregistered with structural data, spatially normalized into the standard MNI space (Montreal Neurological Institute), then, outliers were detected (ART-based scrubbing) and finally images were smoothed using a Gaussian kernel of 8 mm FWMH. All preprocessing steps were conducted using default preprocessing pipeline for volume-based analysis (to MNI-space). As recommended, band-pass filtering was performed with a frequency window of 0.008 to 0.09 Hz (Weissenbacher et al. 2009). Then, structural data were segmented in GM, WM and cerebrospinal fluid and normalized in the same default preprocessing pipeline. Whole-brain analysis was performed using Region of Interest (ROI-to-ROI) approach according to Conn toolbox options, and previously used in a recent study (Demirakca et al. 2015). In order to get a complete picture of possible cerebral changes, we used all existing areas as ROIs, based on the pre-defined ROIs loaded automatically in Conn toolbox, including default network connectivity (FOX) and a complete list of Brodmann areas obtained from the Talairach Daemon atlas (Lancaster et al. 2000). Following recommendations, *p*-FDR threshold was used in the connection-level analysis to correct for multiple comparisons (Whitfield-Gabrieli and Nieto-Castanon 2012). Baseline differences in brain connectivity values between the REHACOP group and CG were introduced as covariates in the interaction analysis (group x time).

#### Memory fMRI paradigm

FMRI data were analyzed using SPM8 (Ashburner et al. 2012). The functional data of each participant were motion-corrected, realigned to the first acquired volume in the session, and a mean realigned volume was created for each participant. Then, all realigned volumes were spatially normalized into the standard MNI space and smoothed using a Gaussian kernel of 8 mm FWMH. Statistical parametric maps were calculated at first-level analysis for each subject with a general linear model, and parameters for the memory fMRI paradigm model specification were introduced. Then, after model estimation, a matrix was obtained for each subject showing higher brain activation while the activation condition compared to the control condition (activation > control).

### Statistical analysis

Demographic, clinical and behavioral variables were analyzed with SPSS (IBM SPSS Statistics 22). Differences between groups were tested with Mann-Whitney U Test and chi-squared test for non-parametric variables. Longitudinal changes between groups in behavioral variables were tested with repeated measures ANOVA 2 × 2 for group x time interaction analysis.

For neuroimaging analysis, whole-brain analysis was performed to study structural and functional cerebral changes. Baseline differences between groups were tested with two-sample t-test analysis. Longitudinal analysis to test differences between pre-treatment and post-treatment for REHACOP group and CG were assessed with repeated-measures ANOVA 2 × 2 analysis data for group x time interaction analysis. The between-subjects factor was group (REHACOP group or CG) and the within-subjects factor was time (pre-treatment and post-treatment). Paired-t-test analysis was also performed to explore intragroup changes. VBM and cortical thickness analyses used total intracranial volume as a covariate. For the fMRI analyses, LEDD was used as a covariate because of the influence of dopaminergic treatment on brain activation (Mattay et al. 2002). Moreover, because the REHACOP group showed lower scores on UPDRS III and higher scores on MMSE at baseline, both variables were included as covariates in longitudinal analyses. For both structural and functional analyses the statistical threshold was set at *p* < .05 corrected for multiple comparisons and *p* < .001 uncorrected analysis was also performed for exploratory results. Effect sizes for each cluster were calculated according to Cohen’s *d* formula (Thalheimer and Cook 2002). Cohen’s *d* statistics of 0.20, 0.50 and 0.80 were considered small, medium and large, respectively (Hojat and Xu 2004). Finally, Rho-Spearman test was used to determine the relationships between MRI data at post-treatment and the performance in cognitive domains after rehabilitation, including executive functions, processing speed, verbal and visual memory and theory of mind; see previous publication (Pena et al. 2014). Bootstrapping was used in correlations to obtain more adjusted results (Efron and Tibshirani 1994).

## Results

### Sociodemographic, clinical characteristics and behavioral data

The sociodemographic characteristics of the sample are shown in Table 1. At baseline, no significant differences were found between groups in age, gender, years of education and clinical aspects of the disease (see Table 1). Regarding behavioral data from the memory fMRI paradigm, no baseline differences were found in hits, correct rejections, false positives or false negatives between groups (Table 1) and no significant changes were found after three months treatment between groups.

### GM volume, cortical thickness and WM indexes

No baseline differences in GM volume, WM indexes or mean cortical thickness (left and right) were found between groups. Longitudinal analysis showed no significant structural changes within or between groups at post-treatment.

### Resting-state fMRI

**Fig. 2:**
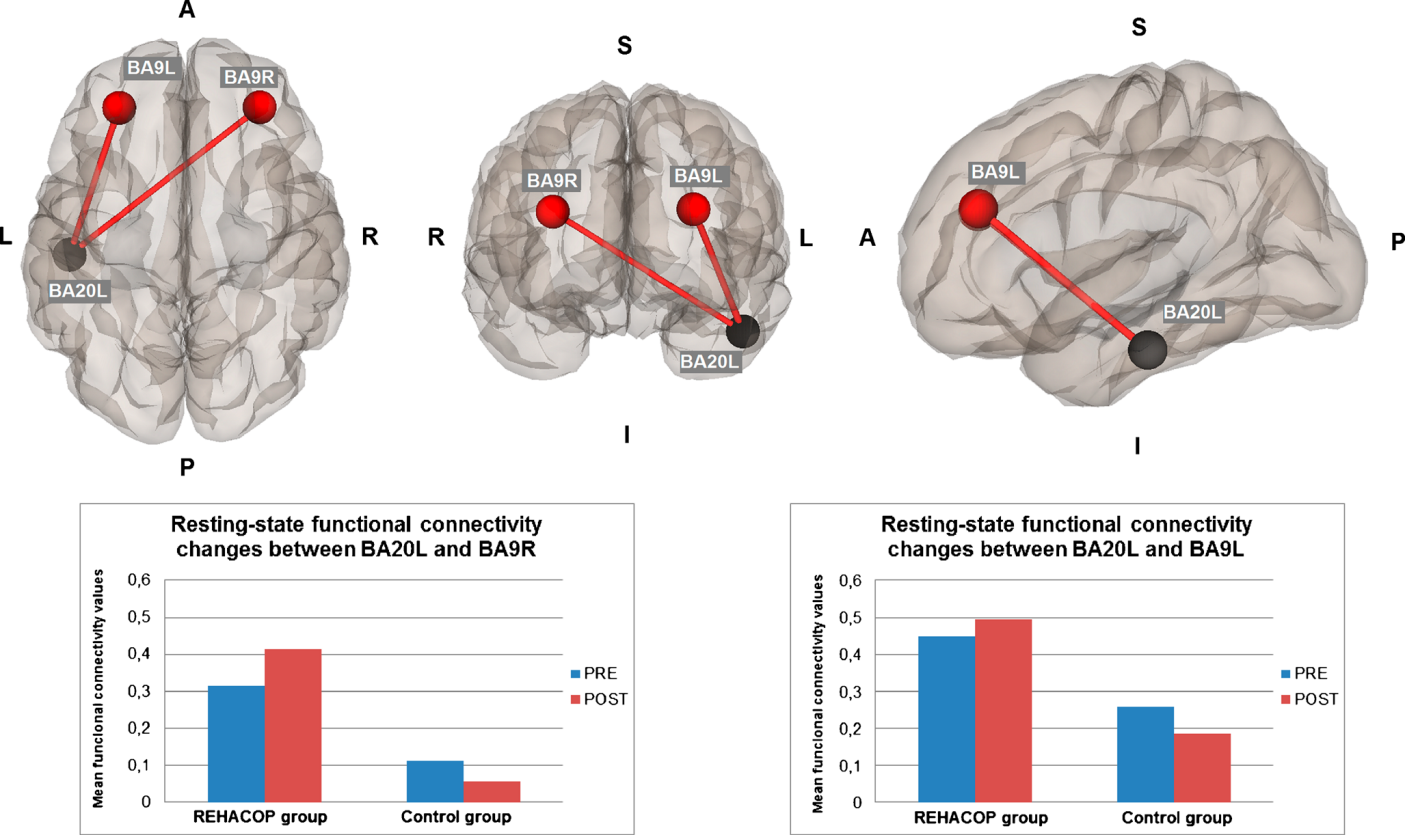
Resting-state brain connectivity fMRI changes (interaction level group x time). Seed (black point) = the left inferior temporal lobe (BA20L; x = −51; y = −23; z = −29); Targets (red points) = left and right dorsolateral prefrontal cortex (BA9L; x = −29; y = 41; z = 25) and (BA9R; x = 33; y = 42; z = 24). Lines represent increased connectivity between the seed and target at the interaction level (group x time), showing the REHACOP group increased brain connectivity at post-treatment compared to the CG. Graphic shows mean connectivity values during resting-state at pre-treatment and post-treatment for REHACOP group and CG. Results are shown at *p* < .05 FDR-corrected. A = Anterior; P = Posterior; I = Inferior; S = Superior; CG = Control Group

Baseline differences in brain activation in resting-state fMRI were found between groups, showing the CG more connectivity between the left dorsal posterior cingulate cortex Brodmann Area (BA31) and the left piriform cortex (BA27) compared to the REHACOP group (*t* = 3.96; *p* = 0.04 FDR-corrected). After controlling for baseline differences, resting-state fMRI data showed significant differences between groups (interaction effect group x time) in functional connectivity between the left inferior temporal lobe (BA20L; x = −51; y = −23; z = −29) and the left and right dorsolateral prefrontal cortex (BA9L; x = −29; y = 41; z = 25; F = 10.71; *p* = .03; *d* = 1.17) and (BA9R; x = 33; y = 42; z = 24; F = 10.01; *p* = .03; *d* = 1.13) respectively, showing the REHACOP group higher brain connectivity at post-treatment compared to the CG (see Fig. 2).

### Memory fMRI paradigm

**Fig. 3:**
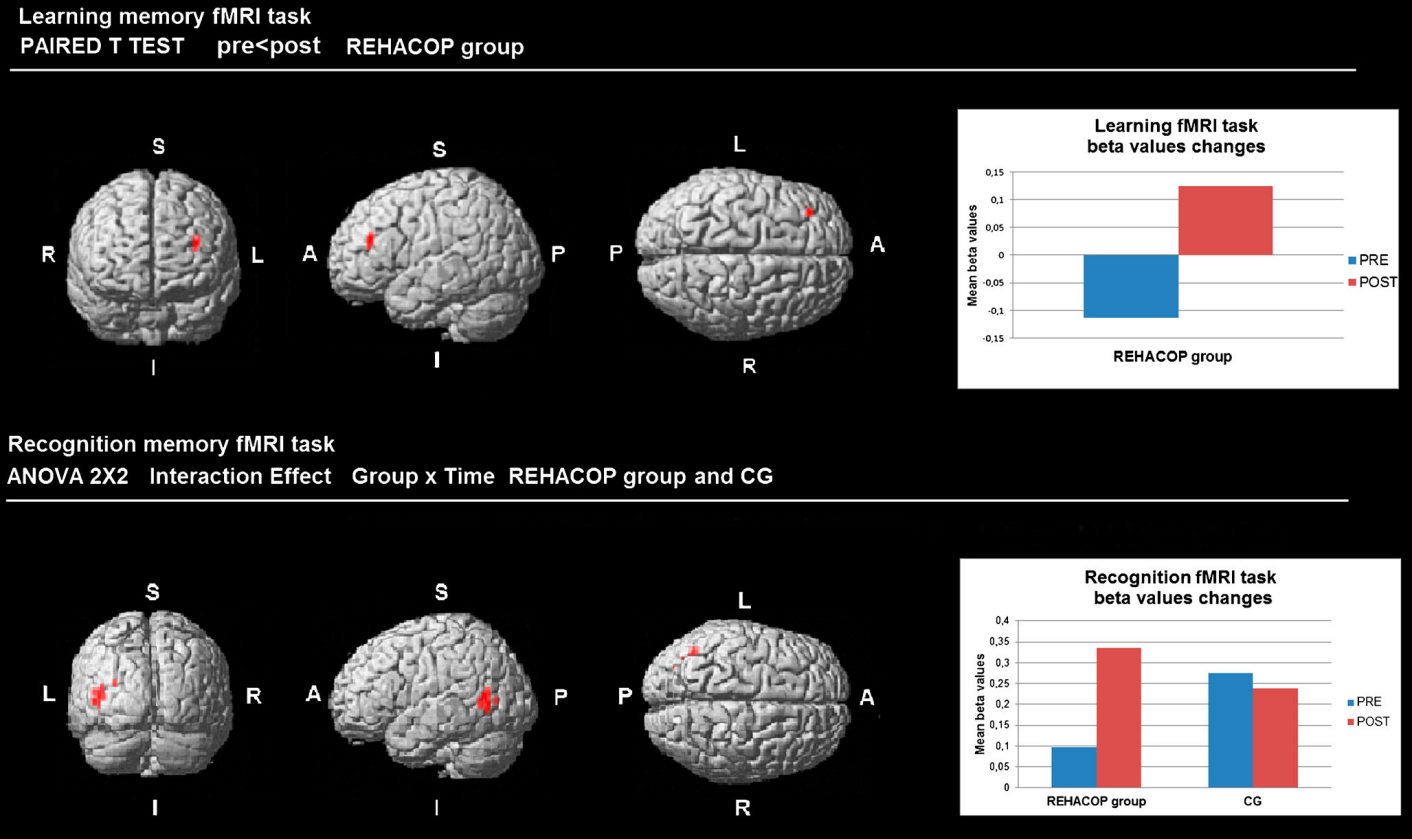
fMRI activation changes during Memory fMRI Paradigm. Areas of brain activation change are shown in red. Graphics show mean beta values while the learning and the recognition memory fMRI tasks at pre-treatment and post-treatment. Results are shown at *p* < .001-uncorrected. A = Anterior; P = Posterior; I = Inferior; S = Superior; CG = Control Group

No baseline differences were found during the learning or the recognition memory fMRI tasks between groups. During the learning memory fMRI task, no significant results were found at the interaction level, but intragroup analysis showed that the REHACOP group increased brain activation in the left frontal lobe at post-treatment compared to pre-treatment (*p* < .001 uncorrected) (see Fig. 3; Table 2). On the contrary, CG showed no significant cerebral changes during the learning memory fMRI task.

**Table 2:** Memory fMRI Paradigm activation changes

	**Cluster size (voxels)**	**MNI coordinate**			**Statistical value**	**Effect size**
		**x**	**y**	**z**		
**Learning memory fMRI Task**						
REHACOP group (pre < post)						
L Frontal Inferior (Pars triangularis)	12	-36	37	22	t = 6.07*	2.21
**Recognition memory fMRI Task**						
Interaction effect (group x time)						
L Middle Temporal Lobe	15	-41	-64	7	F = 30.40*	2.08

Cluster size denotes the extent of the cluster of significant voxels. MNI coordinates refer to the location of the most statistically significant voxel in the cluster. Effect sizes were calculated with Cohen’s d.

*L* Left, *MNI* Montreal Neurological Institute

*Differences are significant at *p* < .001-uncorrected

During the recognition memory fMRI task, repeated measures analysis (interaction effect group x time) revealed significant brain activation changes at post-treatment in the left middle temporal lobe in the REHACOP group compared to the CG (*p* < .05 FWE-corrected). Only few voxels survived the corrected level, hence, results at *p* < .001 uncorrected are showed in Fig. 3 and Table 2.

### Correlations between MRI data and neuropsychological scores in the REHACOP group at post-treatment

Results showed that the brain connectivity between the left inferior temporal lobe and the left dorsolateral prefrontal cortex during resting-state fMRI correlated with the performance on executive functions at post-treatment (Rho = .574; 95 % Confidence Interval [CI] = .083–.842; Standard Error [SE] = .178; *p* = .032). In addition, after cognitive rehabilitation, the REHACOP group showed a significant correlation between the brain activation during learning fMRI task and the scores on visual memory (Rho = .596; CI = .001–.950; SE = .263; *p* = .025). Finally, a marginally significant correlation was found between the brain activation during the recognition fMRI task and the performance on verbal memory at post-treatment (Rho = .512; CI = −.053–.824; SE = .224; *p* = .060).

## Discussion

The objective of this study was to assess cerebral changes related to the integrative cognitive rehabilitation program REHACOP in patients with PD. These results show that patients with PD attending REHACOP program increased their brain connectivity between the temporal and bilateral frontal lobes during resting-state fMRI and increased brain activation in the frontal and temporal lobes during a memory fMRI paradigm. Moreover, the brain connectivity and activation in the REHACOP group at post-treatment correlated with the final performance in cognitive functions. Findings suggest the existence of brain plasticity in patients with this pathology, despite the neurodegenerative process, and support the efficacy of cognitive rehabilitation treatments on PD.

PD patients that received cognitive rehabilitation showed increased brain connectivity between the left inferior temporal lobe and the bilateral dorsolateral prefrontal cortex. Recently, reduced connectivity in the fronto-temporal network has also been found in PD and has been related to working memory encoding deficits in the disease (Wiesman et al. 2016). Impairment in the fronto-temporal network has also been found in schizophrenia patients, and are suggested to underlie encoding deficits (Wolf et al. 2007). In addition, the greater connectivity between temporal and dorsolateral prefrontal cortex has been related with the better performance in word recognition in healthy controls (Wolf et al. 2007). Moreover in this study, the cognitive function of attention was trained during 4 weeks and interestingly, a previous resting-state fMRI study in PD patients also found increased brain connectivity in the dorsolateral prefrontal cortex after attention rehabilitation (Cerasa et al. 2014). Furthermore, the fronto-temporal network connects the prefrontal with the temporal cortex, both areas related to other cognitive functions trained during the REHACOP program, such as executive functions (Nagano-Saito et al. 2005), language, verbal fluency (Pereira et al. 2009), memory (Cabeza and Nyberg 2000; van Paasschen et al. 2013) and theory of mind (Díez-Cirarda et al. 2015).

Results also showed that REHACOP group had increased brain activation after cognitive rehabilitation during the learning and recognition tasks of the memory fMRI paradigm. Specifically, during the recognition fMRI task, the REHACOP group showed increased brain activation in the left middle temporal lobe at post-treatment compared to the CG. These findings confirm previous studies that related the temporal lobe to the retrieval process (Cabeza and Nyberg 2000). Furthermore, during the learning fMRI task, PD patients from the REHACOP group had increased brain activation in the left inferior frontal area at post-treatment compared to pre-treatment. These results are coherent with previous literature because the frontal lobe is known to be involved in memory performance in PD in both encoding and retrieval processes (Cabeza and Nyberg 2000; Eichenbaum et al. 2007). However, the brain activation changes during memory fMRI paradigm should be taken with caution because they were found at an uncorrected level *p* < .001. Increased activation in the frontal and temporal areas after memory rehabilitation has also been found in multiple sclerosis (Chiaravalloti et al. 2012), mild cognitive impairment (Belleville et al. 2011) and healthy adults (Belleville et al. 2011). Compared to PD patients in this study, Alzheimer’s disease patients showed activation changes in frontal but not temporal areas during a recognition fMRI task after memory rehabilitation (van Paasschen et al. 2013). Some authors suggested that Alzheimer’s disease patients could compensate the more pronounced degeneration of the temporal lobe with an overactivation of the frontal lobe (Schwindt and Black 2009). Interestingly, the cerebral changes found during memory fMRI paradigm in this study were located in the left hemisphere, and verbal memory is known to be (in most cases) a cognitive function lateralized in the left hemisphere (Kelley et al. 1998).

Brain activation changes in the REHACOP group cannot be related to the treatment duration or to the format (group vs. individual) because the CG received occupational therapy with the same frequency, duration, and group format. Moreover, brain changes cannot be related to learning effects in the memory fMRI paradigm because different versions were used at pre-treatment and post-treatment.

With all, these findings suggest that integrative cognitive rehabilitation programs have an impact on cerebral activation and connectivity in PD patients. In addition, significant and positive relationships between the brain connectivity and activation and cognitive performance have been found in the REHACOP group after attending cognitive rehabilitation. These findings may suggest that the brain changes increased the activity which helped patients during cognitive performance. Findings of the present study go in line with previous research in other pathologies that also found improvements in cognitive functions and increased brain activation after cognitive rehabilitation (Belleville et al. 2011; Cerasa et al. 2014; Chiaravalloti et al. 2012; van Paasschen et al. 2013). However, decreased brain activation has also been related to better cognitive performance after training in PD (Nombela et al. 2011).

This study also assessed whether cognitive rehabilitation programs could be related to GM changes. As expected by the authors, no significant differences in GM volume after three months of cognitive rehabilitation were found. A previous study with multiple sclerosis patients who received cognitive treatment for the same period of time as in the present study, found the same negative findings (Filippi et al. 2012). Contrary to these results, schizophrenia patients showed neuroprotective effects against GM loss related to a two year intensive cognitive rehabilitation program (Eack et al. 2010) (60 h/week neurocognitive rehabilitation plus 45 weekly social/cognitive group sessions). Similarly, studies in healthy participants showed GM volume changes after three months of intensive cognitive activity (Draganski et al. 2006) and cortical thickness changes after memory training (Engvig et al. 2010). Furthermore, this study found no significant changes in WM integrity and diffusivity after REHACOP program. Filippi et al. (2012) found the same negative findings in multiple sclerosis patients in the assessment of WM volume and diffusivity changes after cognitive rehabilitation. On the contrary, Penadés et al. (2013) found increased FA after four months of cognitive rehabilitation in schizophrenia patients. Therefore, the neurodegenerative process itself and the intensity of the cognitive program might be important variables to understand the absence of GM and WM changes in PD patients of this study. Findings of this study suggest that after three months of an integrative cognitive rehabilitation program, brain activation and connectivity changes could be found in PD, but these functional changes are not accompanied by structural changes.

Several limitations of this study must be taken into account. First, the sample size is small. However, despite the reduced sample size, both groups were equivalent in sociodemographic and clinical variables at baseline, and results showed consistent changes in brain activation values. All significant results showed large effect sizes, which support the clinical relevance of the findings (Hojat and Xu 2004). Future studies with larger samples are needed to replicate these findings in PD. Furthermore, longitudinal follow-up studies must be carried out to evaluate the course of brain changes after cognitive treatments. Moreover, it would be interesting to assess functional brain activation changes during other cognitive tasks, such as executive functions, processing speed or visuo-constructive abilities. Finally, PD patients were mainly at first Hoehn and Yahr stages of the disease. Therefore, further studies with PD patients at moderate and severe stages are needed to evaluate whether these findings can be replicated in more advanced stages of the disease.

## Conclusions

In conclusion, this study reported increased brain activation and connectivity in PD patients after attending an integrative cognitive rehabilitation program. This study, together with results from previous research, adds evidence of the neurobiological effects of cognitive rehabilitation programs in patients with PD.
